# TOPK Drives IL19-Mediated Crosstalk Between Cancer Cells and Fibroblasts to Promote Solar UV-Induced Skin Damage and Carcinogenesis

**DOI:** 10.3390/cancers17132067

**Published:** 2025-06-20

**Authors:** Asad U. Khan, Qiushi Wang, Eunmiri Roh, Sally E. Dickinson, Georg T. Wondrak, Clara Curiel-Lewandowski, Ann M. Bode, Tianshun Zhang

**Affiliations:** 1 The Hormel Institute, University of Minnesota, 801 16th Ave NE, Austin, MN 55912, USA; khan0826@umn.edu (A.U.K.); wang5719@umn.edu (Q.W.); roheunmiri@kwu.ac.kr (E.R.); bodex008@umn.edu (A.M.B.); 2 Department of Cosmetic Science, Kwangju Women’s University, Gwangju 62396, Republic of Korea; 3 Department of Pharmacology, College of Medicine, University of Arizona Cancer Center, Skin Cancer Institute, University of Arizona, Tucson, AZ 85724, USA; sdickinson@uacc.arizona.edu (S.E.D.); wondrak@arizona.edu (G.T.W.); ccuriel@arizona.edu (C.C.-L.); 4 Department of Pharmacology and Toxicology, R Ken Coit College of Pharmacy, University of Arizona, Tucson, AZ 85724, USA; 5 Dermatology Division, College of Medicine-Tucson, University of Arizona, Tucson, AZ 85724, USA

**Keywords:** TOPK, RNAseq, IL19, keratinocytes, cSCC, fibroblast

## Abstract

**Background/Objectives:** Non-melanoma skin cancer (NMSC) is among the most common cancers in the United States, with solar ultraviolet (UV) radiation being a primary etiological factor. T-LAK cell-originated protein kinase (TOPK), a serine/threonine kinase activated by solar UV, has been implicated in skin carcinogenesis. This study aimed to investigate the mechanistic role of TOPK in solar UV-induced skin damage and tumor development. **Methods:** RNA sequencing (RNA-seq) was performed on skin tissues from wild-type (WT) and TOPK knockout (KO) mice, with or without solar UV exposure, to identify TOPK-regulated genes and pathways. Follow-up experiments using Western blotting, immunofluorescence, and luciferase assays were conducted in vitro and in vivo. Functional assays included 3D spheroid and Transwell co-culture systems involving cutaneous squamous cell carcinoma (cSCC) and fibroblast cells. **Results:** TOPK deletion altered gene expression profiles and inhibited solar UV-induced activation of multiple signaling pathways, including cytokine–cytokine receptor interaction, PI3K/AKT, MAPKs, PKG, cAMP, and calcium signaling. RNA-seq and protein analyses identified interleukin-19 (IL19) as a key downstream effector suppressed by TOPK deletion. In cSCC and fibroblast cells, TOPK knockdown reduced IL19 expression and secretion. IL19 promoted cSCC growth and activated PI3K/AKT, ERK, and TOPK pathways. Additionally, chronic TGFβ exposure increased IL19 expression and activated fibroblasts, as indicated by elevated αSMA and FAPα levels. **Conclusions:** These findings establish TOPK as a central regulator of solar UV-induced skin carcinogenesis, partially via modulation of IL19 signaling and fibroblast activation. Targeting TOPK may offer a novel strategy for the prevention and treatment of NMSC.

## Simple Summary

Non-melanoma skin cancer is one of the most common types of cancer, and its development is closely linked to long-term exposure to sunlight. In this study, we investigated a protein called TOPK, which becomes more active after sun exposure and may play a key role in the development of skin cancer. Using laboratory models and skin cancer cells, we found that removing TOPK reduces sunlight-induced damage and slows cancer growth. We also discovered that TOPK regulates another protein, IL19, which helps cancer cells grow and influences surrounding skin tissue. Our findings suggest that targeting TOPK could be a promising strategy to prevent or treat sun-related skin cancer. This research offers new insights into how skin cancer forms and could help guide the development of future protective strategies.

## 1. Introduction

Non-melanoma skin cancers (NMSCs), encompassing basal cell carcinoma (BCC) and cutaneous squamous cell carcinoma (cSCC), represent the most frequently diagnosed malignancies worldwide [1,2,3], with an estimated 5.4 million cases diagnosed annually in the United States alone [4]. The primary etiological factor for these cancers is solar ultraviolet (UV) radiation, which induces DNA damage and alters cellular signaling pathways, leading to skin damage and carcinogenesis [5,6,7]. Although sun protection is recommended, active prevention interventions are generally not used until the development of actinic keratoses (AKs) or cSCCs. Patients with large burdens of AKs or cSCCs require a combination of therapies and often will try several modalities to find the “best fit” [8,9,10]. Understanding the molecular mechanisms underlying UV-induced skin damage and the progression to NMSC is crucial for developing effective prevention and treatment strategies.

T-LAK cell-originated protein kinase (TOPK), also known as PBK, is a serine/threonine kinase that plays significant roles in cancer process through the mediation of cell proliferation, survival, differentiation, and metastasis [11,12,13,14,15,16,17]. Our recent studies have illuminated the critical function of TOPK in skin cancer development. Targeting TOPK can efficiently suppress solar UV-induced skin carcinogenesis [18,19,20], yet its specific role in UV-induced skin damage and carcinogenesis remains to be fully elucidated. In particular, its role in mediating epidermal–dermal communication and cancer–fibroblast interactions remains largely unexplored.

A key component of the tumor microenvironment (TME) in cSCC is the crosstalk between cancer cells and fibroblasts, which profoundly influences tumor growth, invasion, and resistance to therapy. Fibroblasts, particularly cancer-associated fibroblasts (CAFs), support tumor progression by secreting pro-tumorigenic factors, remodeling the extracellular matrix, and modulating immune responses [21,22,23]. Chronic exposure to inflammatory cytokines can induce fibroblast activation, leading to enhanced tumor cell proliferation and migration [24,25,26]. Additionally, fibroblast-derived factors can reciprocally activate oncogenic signaling pathways in cancer cells [24,27], creating a feedforward loop that drives malignancy. Despite increasing recognition of the role of fibroblasts in skin cancer, the molecular mechanisms governing this interaction remain poorly understood. Interleukin-19 (IL19), a member of the IL-10 cytokine family, is an emerging immunomodulatory molecule implicated in inflammatory diseases and cancer [28,29]. IL19 is primarily produced by keratinocytes, immune cells, and fibroblasts in response to inflammatory stimuli [30,31]. Research evidence suggests that IL19 plays a crucial role in increasing growth factor expression, influencing both epidermal and dermal compartments in skin [31].

This study aims to elucidate the mechanistic role of TOPK in solar UV-induced skin carcinogenesis, with a particular focus on its regulation of IL19 and its impact on fibroblast activation. By investigating how TOPK influences the dynamic interplay between cSCC cells and the surrounding stroma, this research provides novel insights into the molecular mechanisms underlying skin cancer progression. A deeper understanding of these processes may offer new therapeutic opportunities for targeting TOPK and its downstream pathways to prevent or treat NMSC.

## 2. Materials and Methods

### 2.1. Reagents and Antibodies

Cell culture medium was obtained from Corning (Corning, NY, USA), and fetal bovine serum (FBS, Cat:100-525) was purchased from Omega Scientific (GeminiBio, West Sacramento, CA, USA). The penicillin/streptomycin solution was sourced from Gene Depot (Katy, TX, USA). Essential reagents for molecular biology and buffer preparation, including Tris, NaCl, trichloroacetic acid (TCA), and SDS, were purchased from Sigma-Aldrich (St. Louis, MO, USA). Recombinant human Transforming Growth Factor-β1 (hTGF-β1, 8915LC) was obtained from Cell Signaling Technology (CST, Danvers, MA, USA). Human IL19 protein (10 µg, P7212) was purchased from MedChemExpress (MCE, Princeton, NJ, USA). VitroGel Hydrogel Matrix (VHM01) for 3D cell culture was obtained from The Well Bioscience Inc. (Monmouth Junction, NJ, USA). A panel of total and phosphorylated antibodies was purchased from CST, including p-PI3K p85 (Tyr458/p55 Tyr199, 4228s), p-AKT (Ser473, 9271s), p-ERK (Thr202/Tyr204, 9101s), p-JNK (Thr183/Tyr185, 9251s), p-p38 (Thr180/Tyr182, 4511s), p-PKA (Ser/Thr, 9621s), p-PKC (Ser, 2261s), and p-TOPK (Thr9, 4941s). The cGKIα (F-5) PKG monoclonal antibody (sc-393987) was obtained from Santa Cruz Biotechnology (Dallas, TX, USA). Antibodies for total protein detection were also purchased from CST, including PI3K p110γ (D55D5, 5405s), AKT (C9272s), ERK p44/42 MAPK (9102), JNK (9252), p38 MAPK (D13E1, 8690), PKA C-α (4782), PKCι/C83H11 (2998s), and TOPK (4942). Additional antibodies were obtained from Santa Cruz Biotechnology, including α-smooth muscle actin (αSMA, IA4, sc-32251), fibroblast activation protein α (FAPα, SS-13, sc-100528), TOPK/PBK (A-6, sc-390817), and glyceraldehyde-3-phosphate dehydrogenase (GAPDH, 0411, sc-47724). The IL19 antibody (orb524337) was purchased from Biorbyt (Durham, NC, USA). Alexa Fluor^®^ 594 anti-cytokeratin (pan-reactive, 628606) was obtained from BioLegend (San Diego, CA, USA), and the vimentin antibody (5741s) was sourced from CST. The Nano-Glo^®^ Dual-Luciferase^®^ Reporter Assay kit (NanoDLR™, N1610) was obtained from Promega (Madison, WI, USA). Tumor-promoting phorbol ester 12-O-tetradecanoylphorbol-13-acetate (TPA, 6B7436) was purchased from Sigma-Aldrich.

### 2.2. RNAseq Analysis in a Mouse Model

SKH-1 hairless mice were purchased from Charles River and acclimated for 2 weeks before the study and had free access to food and water. TOPK knockout SKH-1 hairless mice were established as previously described [20]. All animal studies were approved by the University of Minnesota Institutional Animal Care and Use Committee (IACUC; 2205-40061A). The SSL source consisted of UVA-340 lamps that emit both UVA (94.5%) and UVB (5.5%) irradiation and were purchased from Q-Lab Corporation (Cleveland, OH, USA). The UVA-340 lamps provide natural sunlight that includes both UVA and UVB in the critical short wavelength region of 365 nm down to the solar cutoff of 295 nm, with a peak emission at 340 nm. The animals were housed in climate-controlled quarters with a 12 h light/12 h dark cycle. The mice were housed and bred under virus- and pathogen-free conditions. SKH-1 hairless mice were divided into four groups (2 females and 2 males per group): wildtype (WT) and TOPK knockout (KO) exposed or not exposed to solar simulated light (SSL) treatment (1 dose at 149 kJ UVA/m^2^ and 7.2 kJ UVB/m^2^). At 24 h after SSL, mouse skins were collected, and RNA was extracted for library construction and sequencing purposes. RNA extraction (PureLink™ RNA Mini Kit, Invitrogen, Carlsbad, CA, USA), sequencing library, and bioinformatic analysis (LC Sciences, Houston, TX, USA) details are included in Supplementary Methods [32,33,34,35,36,37].

### 2.3. Cell Culture

The human skin keratinocyte (HaCaT), human epidermoid carcinoma A431, BJ skin fibroblasts, and the HEK293T cell line were purchased from American Type Culture Collection (ATCC). The human squamous cell carcinoma SCC-12 cell line was purchased from Thermo Fisher Scientific (Waltham, MA, USA). Normal human dermal fibroblasts (NHDF) were purchased from Lonza (Walkersville, MD, USA). The HaCaT, HEK293T, BJ, NHDF, and A431 cell lines were cultured in Dulbecco’s Modified Eagle’s Medium (DMEM) containing 10% FBS and 1% antibiotics. The SCC-12 cell line was cultured in RPMI 1640 1X medium with L-glutamine supplemented with 10% FBS, 1% antibiotics, and 1% MEM non-essential amino acid solution. All cell culture conditions were conducted according to ATCC or Lonza’s instructions.

### 2.4. Western Blot Analysis

Western blotting was conducted following established protocols described previously [38]. The extracted proteins were prepared for Western blot analysis, and detailed information is described in Supplementary Methods. Primary antibodies were diluted 1:1000 and incubated overnight at 4 °C, followed by incubation with an HRP-conjugated secondary antibody diluted 1:10,000. Protein bands were visualized using a chemiluminescent substrate (GE Healthcare Biosciences, Piscataway, NJ, USA).

### 2.5. Lentiviral Infection

For lentiviral infection, lentivirus plasmids shTOPK (#1, TRCN0000001807; 5′ CCGGGAATATGGCAAGAGGGTTAAACTCGAGTTTAACCCTCTTGCCATATTCTTTTT-3′,#2TRCN0000001806;5′CCGGCACCAAGCAAATTATCAGAAACTCGAGTTTCTGATAATTTGCTTGGTGTTTTT -3′) were purchased from GE Healthcare Dharmacon (Open Biosystems, Marlborough, MA, USA). A pLKO.1-puro non-targeting shRNA control plasmid was acquired from Sigma-Aldrich (St. Louis, MO, USA). For lentivirus production, HEK293T cells were transfected with either shTOPK or control plasmid along with the packaging plasmids pMD2.G and psPAX (Thermo Scientific, Huntsville, AL, USA) using iMFectin poly DNA transfection reagent (GenDEPOT, Barker, TX, USA) following the manufacturer’s protocol. Viral supernatant fractions were collected at 24 and 48 h post-transfection, filtered through a 0.45 μm syringe filter, and used to infect HaCaT, A431, SCC-12 cells, and BJ and NHDF fibroblasts in the presence of 10 μg/mL polybrene (Millipore, Burlington, MA, USA). After 24 h, the medium was refreshed, and the cells were subjected to puromycin selection (2 μg/mL) for 48 h. Selected cells were expanded and utilized for subsequent experiments.

### 2.6. MTS and Crystal Violet Assays

To evaluate the effect of IL19 on cancer cell growth, A431 and SCC-12 cells (2 × 10^3^ cells per well) were seeded in 96-well plates with complete growth medium and incubated overnight. The following day, cells were treated with IL19 at concentrations of 20, 40, and 80 ng/mL in serum-free medium and incubated for five days. On day 5, the MTS reagent was added for 1 h, and absorbance was measured at 492 nm using the Luminoskan Ascent and Multiskan MCC (LabSystems, Helsinki, Finland). Additionally, a crystal violet assay was performed to assess cell growth.

TGFβ promotes the transition of fibroblasts into cancer-associated fibroblasts (CAFs) [39]. To generate activated CAF-like fibroblasts, BJ and NHDF fibroblasts were treated with TGFβ (10 ng/mL) for two weeks. shTOPK and shControl BJ-TGFβ and NHDF-TGFβ cells were generated. Once the cells reached 70–80% confluency, they were cultured in serum-free media to generate conditioned media (CM). The collected CM was then used to treat cSCC cells (A431 or SCC-12) for 0, 24, 48, and 72 h, with the medium replaced every 24 h. The MTS assay was performed to measure absorbance at 492 nm, and crystal violet staining was conducted at the final time point (72 h). All experiments were performed in triplicate, and a blank sample (a well containing medium and reagent but no cells) was included as a reference control on each plate to correct for background absorbance.

### 2.7. Luciferase Reporter Assay

HaCaT, A431, SCC-12, BJ, NHDF, BJ-TGFβ, or NHDF-TGFβ cells were seeded in 12-well plates (1 × 10^5^ cells per well). Stable control and TOPK knockdown cells were co-transfected with 50 ng of the NF-κB luciferase reporter plasmid and 20 ng of the β-galactosidase plasmid as an internal control, using the iMFectin poly DNA transfection reagent (GenDEPOT) according to the manufacturer’s instructions. After 24 h of transfection, the medium was replaced with 1 mL fresh medium per well. HaCaT cells were then exposed to solar-simulated light (SSL) at 60 kJ UVA/m^2^ and 2.9 kJ UVB/m^2^. A431 and SCC-12 cells were treated with or without 20 ng/µL TPA. Following treatment of HaCaT and cSCC cells and fibroblasts, the medium was removed, and each well was rinsed with 1× PBS. Then, 150 µL of luciferase lysis buffer was added to each well. Luciferase and β-galactosidase activities were measured using the BioTek SYNERGY Neo2 multi-mode reader (BioTek Instruments, Winooski, VT, USA) and the Luminoskan Ascent and Multiskan MCC (Lab Systems), respectively. Luciferase activity was normalized to β-galactosidase activity for each cell line. All experiments were performed in triplicate.

### 2.8. Immunofluorescence

HaCaT cells (5 × 10^4^ per well) were cultured in chamber slides and incubated overnight at 37 °C in a 5% CO_2_ humidified incubator. The next day, the medium was replaced with fresh medium, and the cells were exposed to SSL at 60 kJ UVA/m^2^ and 2.9 kJ UVB/m^2^. A431 cells (5 × 10^4^ per well) were similarly seeded in chamber slides and incubated overnight. The next day, fresh medium with or without 20 ng/µL TPA was added, and the slides were incubated at 37 °C in a 5% CO_2_ humidified incubator for 16 h. BJ, NHDF, BJ-TGFβ, and NHDF-TGFβ fibroblasts (5 × 10^4^ per well) were treated with 80 ng/mL IL19 in serum-free medium for 16 h. After treatment, all cells were washed twice with PBS and fixed with 100% chilled methanol for 10 min at −20 °C, followed by rinsing twice with PBS containing 0.05% Tween-20 (PBS-Tw). Cells were then blocked with PBS containing 10 mg/mL BSA for 30 min at room temperature on a shaker before incubation with primary antibodies overnight at 4 °C: NF-κB (p65, D14E12 [XP] rabbit mAb, 1:200), alpha-tubulin (SC-69969, mouse mAb, 1:500), and α-SMA (sc-32251, 1:500). The next day, slides were washed 3 times for 10 min each with PBS-Tw, followed by incubation with 500 µL of 2% goat serum in PBS-BSA for 20 min. Secondary antibodies, goat anti-rabbit Alexa Fluor 488 (1:1000) and goat anti-mouse Alexa Fluor 594 (1:1000), were then applied for 45 min at room temperature in the dark. Slides were washed again 3 times with PBS-Tw for 10 min each, excess buffer was removed, and one drop of Fluoro-Gel II with DAPI was added before mounting coverslips and sealing with nail polish.

### 2.9. Spheroid 3D Co-Culture

VitroGel Hydrogel Matrix was used for 3D cell culture in this experiment following the manufacturer’s instructions, using a 48-well plate for the setup. Briefly, ShCon and shTOPK BJ or BJ-TGFβ fibroblasts (2 × 10^4^ cells each) and cSCC cells (A431, SCC12; 2 × 10^4^ cells each) were mixed with 200 µL of hydrogel and added to each well. The plate was kept at room temperature for 15 min to allow gelation. Subsequently, 200 µL of culture medium was added to each well, and the plate was incubated overnight at 37 °C in a 5% CO_2_ humidified incubator. After four days, immunofluorescence staining was conducted following an established protocol (Protocol_Immunofluoroscence-Staining.pdf). For primary antibodies, vimentin (D21H3) XP rabbit mAb (Cell Signaling Technology, Cat. No: 5741, 1:100 dilution) and Alexa Fluor^®^ 594 anti-cytokeratin (BioLegend, Cat. No: 628606, 1:100 dilution) were used, and the plate was incubated overnight at 4 °C.

### 2.10. Migration Assay in Transwell Co-Culture System

To assess the role of fibroblasts in promoting cancer cell growth, a migration assay was conducted using a Corning 24-well plate with 6.5 mm Transwell inserts containing an 8.0 µm pore polycarbonate membrane. A431 cells (3 × 10^4^ cells per well) were seeded in the upper membrane insert with serum-free medium, while fibroblasts (BJ shCon, shCon, BJ-TGFβ shCon, BJ-TGFβ shTOPK#1, and BJ-TGFβ shTOPK#2; 5 × 10^4^ cells per well) were seeded in the lower chamber. The plate was incubated in serum-free medium for 96 h. Following incubation, crystal violet staining was performed, and images were captured using an Olympus E-420 digital camera mounted on an Olympus CKX41 inverted phase-contrast microscope. For colony quantification, a 10% acetic acid solution in ddH_2_O was prepared, and 350 µL was added to each membrane insert. Colonies were carefully dissolved using a pipette, and 100 µL of the crystal violet-based solution was transferred to a 96-well plate. The plate was placed on a shaker for 15 min, and absorbance was measured at 490 nm using a Luminoskan Ascent and Multiskan MCC (Lab Systems).

### 2.11. Statistical Analysis

All statistical analyses were performed using IBM SPSS Statistics (version 23). To evaluate differences among experimental groups, data were analyzed by one-way analysis of variance (ANOVA) followed by Dunnett’s post hoc test to compare each experimental group against the control. All data values are represented as mean values ± SD, and *p*-values less than or equal to 0.05 were considered statistically significant. GraphPad Prism version 9 was used for precise statistical analysis and graphical representation.

## 3. Results

### 3.1. TOPK Mediates Solar UV-Induced Changes in Gene Expression Through Multiple Signaling Pathways

**Figure 1:**
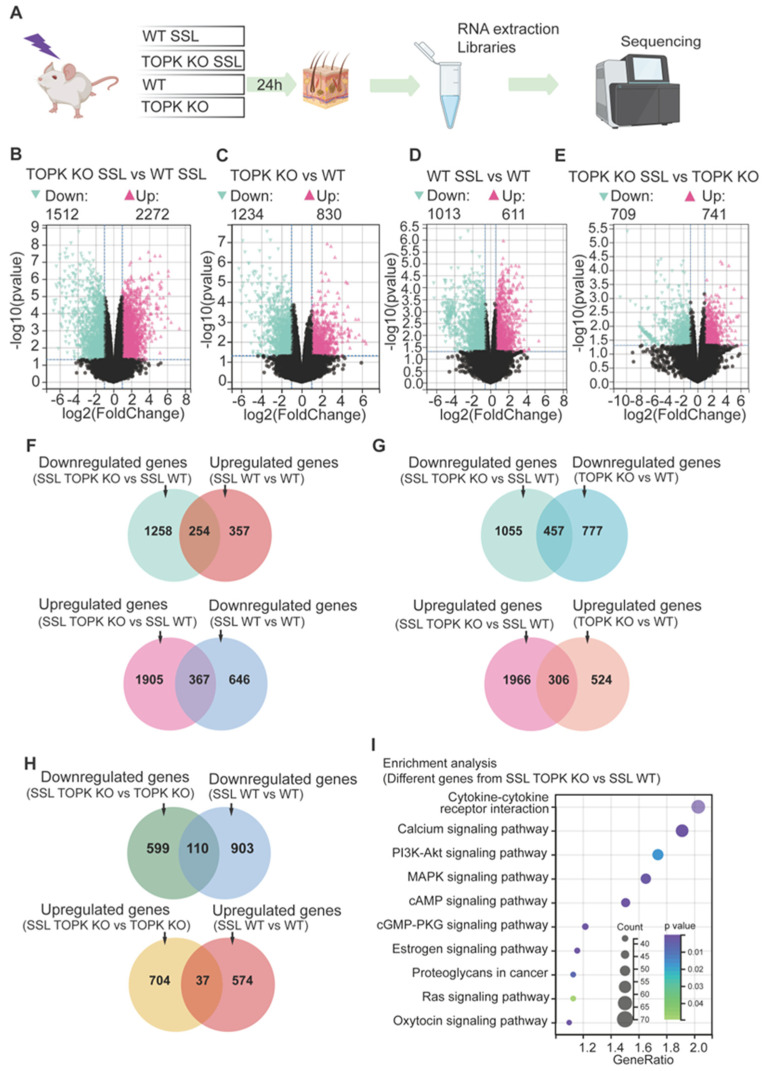
TOPK mediates solar UV-induced gene expression changes through multiple signaling pathways. (**A**) SKH-1 hairless mice were divided into four groups: wildtype (WT) and TOPK knockout (KO) with or without solar simulated light (SSL) treatment. At 24 h after 1 dose of SSL irradiation (149 kJ UVA/m^2^ and 7.2 kJ UVB/m^2^), dorsal mouse skins were collected, and RNA samples were extracted for library construction and sequencing. (**B**–**E**) The R package Limma (version 3.40.6) was used to identify differences in gene expression between groups. To be considered relevant, gene expression had to differ significantly (*p* < 0.05) and exhibit an absolute fold change of ≥2. (**F**) Overlap between genes in SSL-treated TOPK vs. SSL-treated WT groups and genes in SSL-treated WT vs. untreated WT groups. (**G**) Overlap between the genes in SSL-treated TOPK KO vs. SSL-treated WT groups and genes in untreated TOPK KO vs. WT groups. (**H**) Overlap between genes in SSL-treated TOPK KO vs. untreated TOPK groups and genes in the SSL-treated WT vs. untreated WT groups. (**I**) Enrichment analysis revealed that various signaling pathways were associated with the altered genes mediated by TOPK in response to SSL treatment. These pathways include cytokine–cytokine receptor interaction, calcium signaling, MAPK pathway, cAMP pathway, and several other signaling pathways. The size of the circle represents the number of genes affected in the pathway. For example, the cytokine–cytokine receptor interaction pathway had the greatest number of genes affected by TOPK KO with SSL treatment. Also, the darker the color, the lower the *p*-value.

The SKH-1 hairless mice were divided into four groups that included WT (wildtype) or TOPK knockout (KO) treated or not treated with SSL. At 24 h after SSL irradiation, the mouse skins were collected, and RNA was extracted for library construction and sequencing (Figure 1A). The R package Limma (version 3.40.6) was used to identify differences in gene expression between the groups. To be considered relevant, a significance level of *p* < 0.05 and an absolute fold change of ≥2 was required. As might be expected, in the SSL-treated groups, the TOPK KO group exhibited a large number of significant changes in gene expression compared to the SSL-treated WT group, with 1512 downregulated and 2272 upregulated genes (Figure 1B). Additionally, in groups without SSL treatment, TOPK KO induced significant downregulation of 1234 genes and upregulation of 830 genes compared to the WT group (Figure 1C). This suggests that TOPK depletion has a significant effect on gene expression even without SSL treatment. In comparing SSL-treated and untreated WT groups, SSL treatment induced significant downregulation of 1013 genes and upregulation of 611 genes (a total of 1624 gene changes) compared to the WT group without SSL treatment (Figure 1D). In the TOPK KO groups, SSL treatment led to 709 downregulated and 741 upregulated genes (a total of 1450 gene changes) compared to TOPK KO without SSL treatment (Figure 1E). All these findings demonstrate substantial alterations in gene expression between the TOPK KO and WT groups, with or without SSL treatment.

To demonstrate the preventive effect of TOPK KO on SSL-induced gene expression changes, we identified 254 genes (Figure 1F, upper panel) that were both downregulated in the SSL-treated TOPK KO group compared to SSL-treated WT group (data from Figure 1B) and upregulated in the SSL-treated WT group compared to the untreated WT group (data from Figure 1D). Additionally, we found 367 genes (Figure 1F, lower panel) that were upregulated in the SSL-treated TOPK KO group compared to the SSL-treated WT group (data from Figure 1B) and downregulated in the SSL-treated WT group compared to the untreated WT group (data from Figure 1D). The overlapping genes in the upper and lower panels of Figure 1F demonstrate that TOPK KO can block SSL-induced alterations in gene expression. To clearly demonstrate that TOPK KO induces gene expression changes both in the presence and absence of SSL treatment, we identified 457 genes (Figure 1G, upper panel) that were downregulated in the TOPK KO group compared to the WT group. Additionally, we identified 306 genes (Figure 1G, lower panel) that were significantly upregulated by TOPK KO compared to WT, whether with or without SSL treatment. These findings illustrate that TOPK KO not only blocks SSL-induced gene expression changes (Figure 1F) but also consistently alters gene expression irrespective of SSL treatment status. The overlapping genes in Figure 1G demonstrate that TOPK KO affects gene expression alterations not only under SSL treatment conditions but also in the absence of SSL treatment. On the other hand, we identified 110 genes (Figure 1H, upper panel) that overlapped between the downregulated genes in the SSL-treated TOPK KO group compared to the untreated TOPK KO group and the downregulated genes in the SSL-treated WT group compared to the untreated WT group. We also found 37 genes (Figure 1H, lower panel) that overlapped between the upregulated genes in the SSL-treated TOPK KO group compared to the untreated TOPK KO group and the upregulated genes in the SSL-treated WT group compared to the untreated WT group. The 110 genes overlapping in the upper panel were significantly downregulated in both the SSL-treated TOPK KO and WT groups compared to the untreated groups. Additionally, the 37 genes overlapping in the lower panel were significantly upregulated in both the SSL-treated TOPK KO and WT groups compared to the groups without SSL treatment. Detailed information on the data is shown in the Supplementary Data for Figure 1.

Genes that exhibited significant differences (*p* < 0.05; ≥2-fold difference) between the SSL-treated TOPK KO and WT groups (data from Figure 1B) were selected for enrichment analysis (KEGG) using the Database for Annotation, Visualization, and Integrated Discovery (DAVID). Enrichment analysis revealed that various signaling pathways were associated with the altered genes mediated by TOPK in response to SSL treatment. These pathways included cytokine–cytokine receptor interaction, calcium signaling, PI3K-AKT, MAPKs, cAMP, and several other signaling pathways (Figure 1I). Note that the larger the circle, the more genes were affected in the pathway. For example, the cytokine–cytokine receptor interaction pathway had the greatest number of genes affected by SSL and TOPK KO. Also, the darker color, the lower the *p*-value. The results demonstrate that TOPK KO could mediate SSL-induced gene expression alterations through multiple signaling pathways.

### 3.2. Deletion of TOPK Can Potentially Mediate AK and SCC Development Through Multiple Signaling Pathways

**Figure 2:**
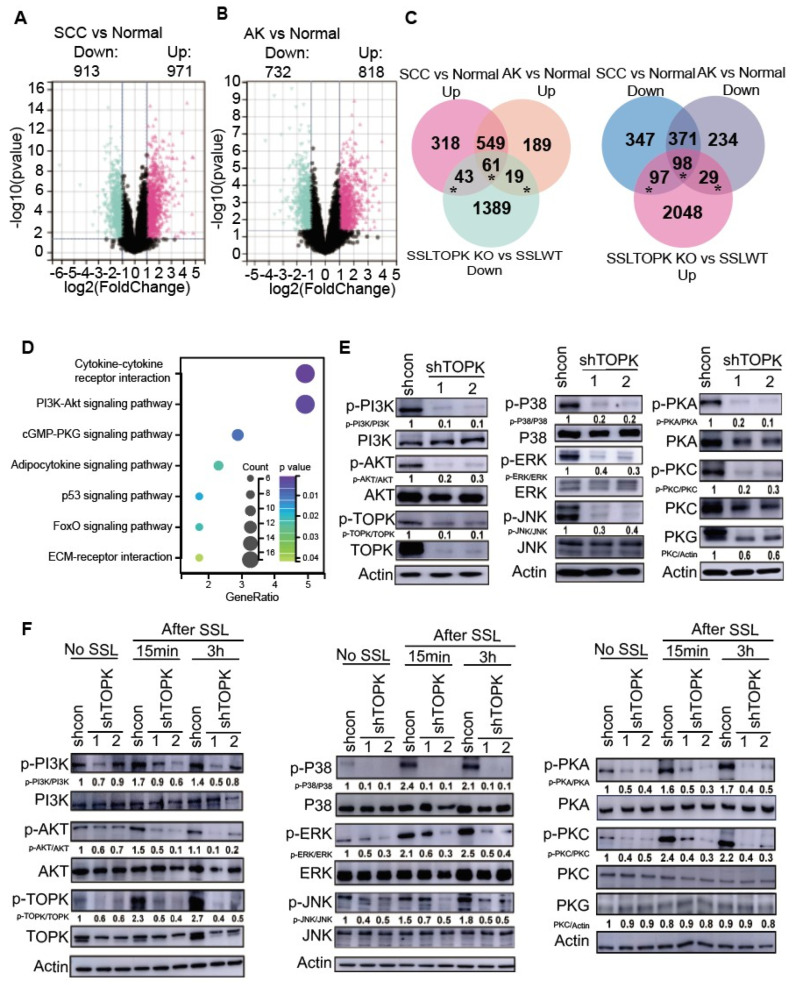
Deletion of TOPK potentially suppresses gene expression changes in AK and SCC. GSE2503, GSE45164, GSE42677, and GSE45216 data were obtained from the GEO database and merged using the R software package in Silico Merging. The samples comprise 55 SCC, 19 AK, and 19 normal tissue specimens. Differences in gene expression between (**A**) SCC vs. normal and (**B**) AK vs normal tissue were assessed. To be considered relevant, gene expression had to differ significantly (*p* < 0.05) and exhibit an absolute fold change of ≥2. (**C**) Overlap between genes in SCC vs. normal, AK vs. normal, and genes in SSL-treated TOPK KO vs. SSL-treated WT groups. The upregulated genes observed in SCC and AK compared with normal tissue overlapped with the downregulated genes in SSL-treated TOPK KO vs. SSL-treated WT groups (**upper panel**). Additionally, the downregulated genes in SCC and AK compared with normal tissue overlapped with the upregulated genes in SSL-treated TOPK KO vs. SSL-treated WT groups (**lower panel**). (**D**) KEGG pathway enrichment analysis demonstrated that multiple signaling pathways are potentially involved in TOPK-mediated AK or SCC development. (**E**) A431 cell lines stably expressing shTOPK or shControl (shCon) were generated, and the phosphorylation and total expression levels of kinases were analyzed by Western blotting. (**F**) Phosphorylation and total expression levels of kinases in HaCaT cells were also assessed by Western blotting. Band intensities were quantified using ImageJ (Version: Fiji 2.16.0 ImageJ).

To investigate the role of TOPK-mediated gene expression changes in human AK and SCC, additional analyses were conducted using data obtained from the GEO database, specifically GSE2503, GSE45164, GSE42677, and GSE45216, which were merged using the R software package in Silico Merging. The samples comprised 55 SCC, 19 AK, and 19 normal tissue specimens. Subsequently, we compared differential gene expression between SCC or AK and normal skin tissue. In SCC vs. normal tissue, 913 genes were downregulated, whereas 971 genes were upregulated (Figure 2A). Additionally, in AK compared with normal tissue, 732 genes were downregulated and 818 genes were upregulated (Figure 2B). Solar UV irradiation is known to be the major cause of AK and SCC. To understand the potential function of TOPK in AK and SCC, the upregulated genes observed in SCC or AK were compared with genes in normal tissue and overlapped with the downregulated genes in the SSL-treated TOPK KO and WT groups (Figure 2C left panel). Additionally, the downregulated genes in SCC or AK were compared with normal tissue and overlapped with the upregulated genes in the SSL-treated TOPK KO and WT groups (Figure 2C right panel). The overlapping genes indicate that deletion of TOPK can potentially prevent gene expression changes in SCC or AK compared to normal tissue. The overlapping genes (marked with an asterisk) were then used for enrichment analysis. Detailed information of the analysis is demonstrated in Supplementary Data for Figure 2. The results demonstrated that the deletion of TOPK has the potential to mediate gene expression changes in AK or SCC through multiple signaling pathways, including the cytokine–cytokine receptor interaction and PI3K-AKT and cGMP-PKG signaling pathways (Figure 2D). Based on results from the RNA sequencing data and data analysis, we performed a series of Western blots to determine and confirm the phosphorylation levels of members of PI3K-AKT, MAPKs and cGMP-PKG signaling pathways in control (shCon) and TOPK knockdown (shTOPK#1 and #2). The phosphorylation levels of signaling pathway members and total PKG level were attenuated by shTOPK in A431 (Figure 2E) and SCC-12 cells (Supplementary Figure S1), respectively. No significant changes were observed in the total protein levels of any signaling pathway members except PKG in cSCC12 cell lines. Our findings revealed that the phosphorylation of signaling pathway members was significantly increased in HaCaT cells exposed to SSL compared to those without SSL exposure, and this increase was inhibited by TOPK knockdown (Figure 2F).

### 3.3. TOPK Drives IL19-Induced cSCC Growth and Fibroblast Activation by Mediating NF-κB Activation

**Figure 3:**
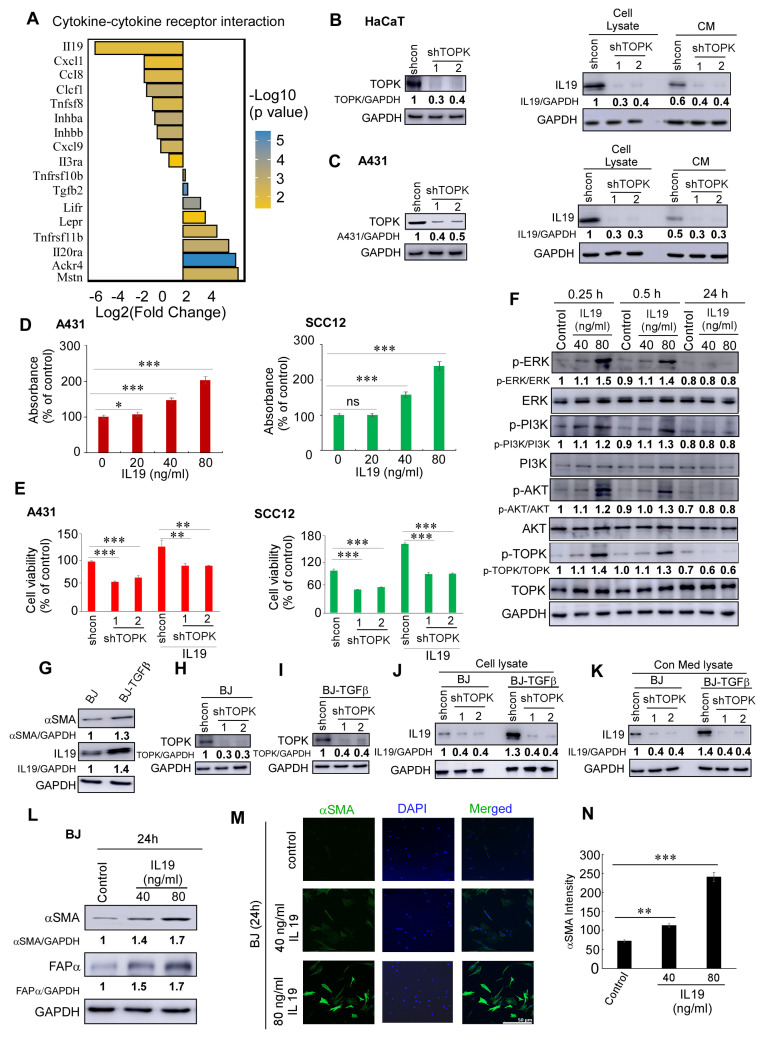
TOPK drives IL19-induced cSCC growth and fibroblast activation. (**A**) Differentially expressed genes in the cytokine–cytokine receptor interaction pathway upon TOPK knockdown. The fold change and *p*-values of key genes are shown, with IL19 exhibiting the most significant decrease. (**B**,**C**) Knockdown of TOPK significantly reduced IL19 expression at the protein level and its secretion into the conditioned media (CM) of keratinocytes and cSCC cells, as determined by Western blotting. (**D**) IL19 treatment promoted cSCC A431 and SCC12 cell growth, as assessed by MTS assay. (**E**) cSCC A431 and SCC12 cells were treated with recombinant human IL19 (40 ng/mL) for 4 days. Cell growth was measured using the MTS assay. IL19 treatment significantly increased cell growth compared to untreated controls. (**F**) IL19 activated ERK, PI3K, AKT, and TOPK phosphorylation within 15 to 30 min of treatment, but this effect was not sustained at 24 h, as shown by Western blot analysis. (**G**) IL19 and αSMA expression were elevated in chronic TGFβ-treated BJ-TGFβ fibroblasts compared with normal BJ fibroblasts. (**H**–**K**) TOPK knockdown significantly suppressed IL19 expression and secretion in both normal BJ fibroblasts and activated BJ-TGFβ fibroblasts. (**L**,**M**) IL19 treatment enhanced the expression of αSMA and FAPα in normal BJ fibroblasts, as demonstrated by Western blot (**L**) and immunofluorescence staining (**M**). (**N**) The density of αSMA was obtained for each sample. Data are shown as means ± SD from three independent experiments. The asterisks indicate a significant difference compared to the group of control samples (*n* = 3) (*, *p* < 0.05; **, *p* < 0.01; ***, *p* < 0.001 and ns: no significant difference, one-way ANOVA followed by Dunnett’s post hoc test).

The fold change and *p*-values of differentially expressed genes in the top pathway from Figure 2D, cytokine–cytokine receptor interaction, are shown in Figure 3A. Among these, IL19 exhibited the most significant change upon TOPK deletion. Furthermore, knockdown of TOPK markedly suppressed IL19 expression in cells and its secretion into the conditioned media (CM) of keratinocytes and cSCC cells (Figure 3B,C). Functionally, IL19 significantly promoted cSCC growth (Figure 3D). Knockdown of TOPK decreased IL19-induced cSCC cell growth (Figure 3E). In addition, IL19 enhanced the phosphorylation levels of ERK, PI3K, AKT, and TOPK within 15 to 30 min after treatment, but not at 24 h post-treatment (Figure 3F).

To mimic cancer-associated fibroblast activation, BJ and NHDF fibroblasts were chronically treated with TGF-β, followed by withdrawal of the treatment. We observed that IL19 and αSMA expression were significantly elevated in activated BJ-TGFβ and NHDF-TGFβ fibroblasts (Figure 3G). Moreover, TOPK knockdown significantly suppressed IL19 expression and secretion in both normal BJ fibroblasts and activated BJ-TGFβ fibroblasts (Figure 3H–K). This finding was further confirmed in normal NHDF fibroblasts and activated NHDF-TGFβ fibroblasts (Supplementary Figure S2). Additionally, IL19 enhanced the expression of αSMA and FAPα in normal BJ fibroblasts, as demonstrated by Western blotting (Figure 3L) and immunofluorescence (Figure 3M,N; Supplementary Figure S3). Quantitative analysis of α-SMA staining (Figure 3N) was performed using fluorescence images acquired with the ZEN Lite software program (version 3.7). Images were captured using an uncompressed format at 100% compression quality to preserve signal integrity. The detector gains for both AF488 and DAPI channels were set at 736 V, with a pixel time of 0.73 μsec and an exposure/frame time of 57.58 s. For image analysis, each microscopic image was opened in 2D view mode. Regions of interest were manually defined using the spline contour drawing tool in the graphics panel, specifically outlining α-SMA–positive areas. After contour selection, the “Measure” function was used to extract area (μm^2^) and fluorescence intensity values from each channel (AF488 for α-SMA and DAPI for nuclei). No arbitrary intensity threshold was applied; instead, mean AF488 fluorescence intensities from all detected α-SMA-positive objects within each image were recorded.

**Figure 4:**
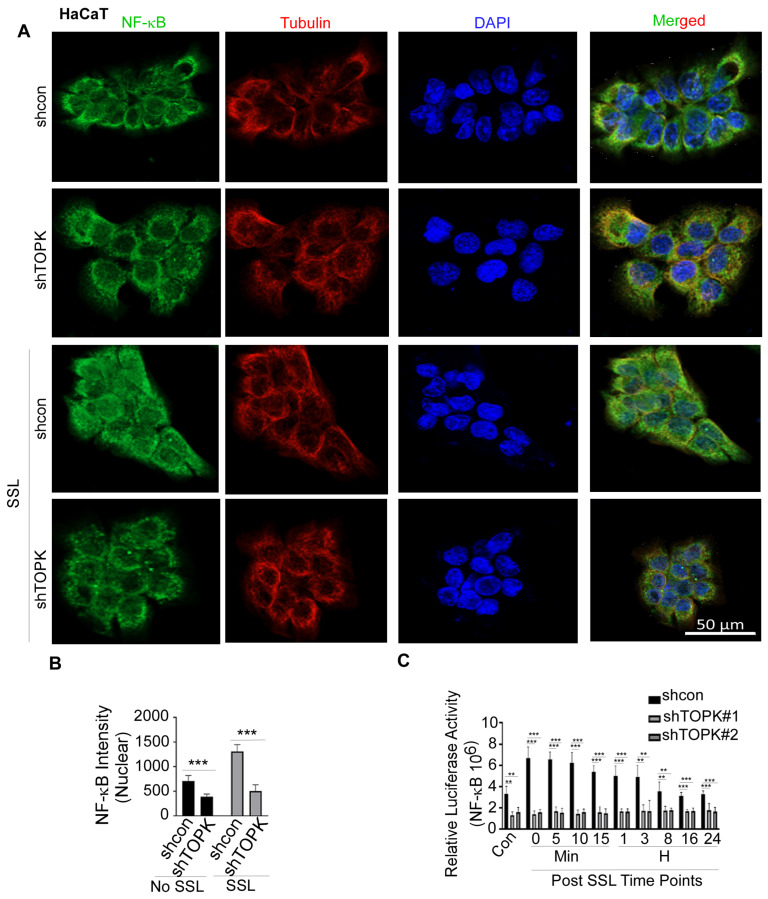
TOPK regulates NF-κB activation and nuclear translocation in HaCat cells. (**A**,**B**) Immunofluorescence analysis shows that TOPK knockdown significantly reduced SSL-induced NF-κB nuclear translocation in keratinocytes. The nuclear density of NF-κB was quantified for each sample. (**C**) TOPK knockdown significantly suppressed SSL-induced NF-κB activation in keratinocytes, with or without SSL treatment, as determined by a luciferase reporter assay. Asterisks indicate significant differences compared to the control group (**, *p* < 0.01; ***, *p* < 0.001; one-way ANOVA followed by Dunnett’s post hoc test).

**Figure 5:**
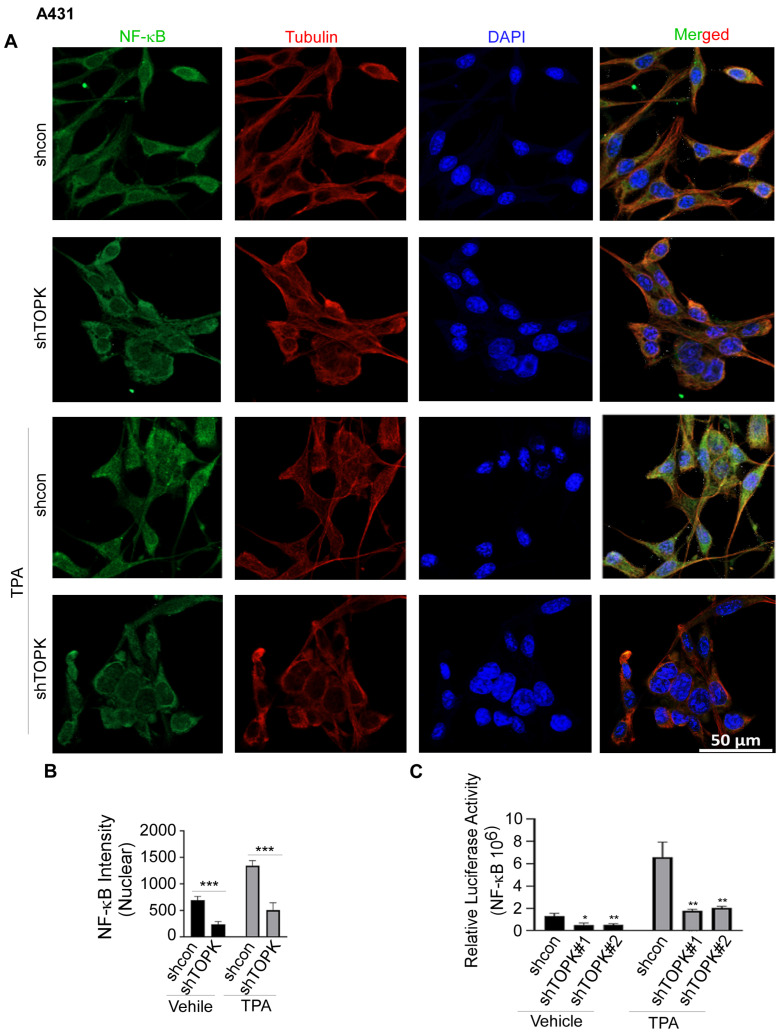
TOPK regulates NF-κB activation and nuclear translocation in A431 cells. (**A**,**B**) Immunofluorescence analysis demonstrates that TOPK knockdown suppressed NF-κB nuclear translocation in A431 cells, both with and without TPA treatment. The nuclear density of NF-κB was quantified for each sample. (**C**) TOPK knockdown markedly decreased NF-κB activation in A431 cells, as measured by a luciferase reporter assay. Asterisks indicate significant differences compared to the control group (*, *p* < 0.05; **, *p* < 0.01; ***, *p* < 0.001; one-way ANOVA followed by Dunnett’s post hoc test).

**Figure 6:**
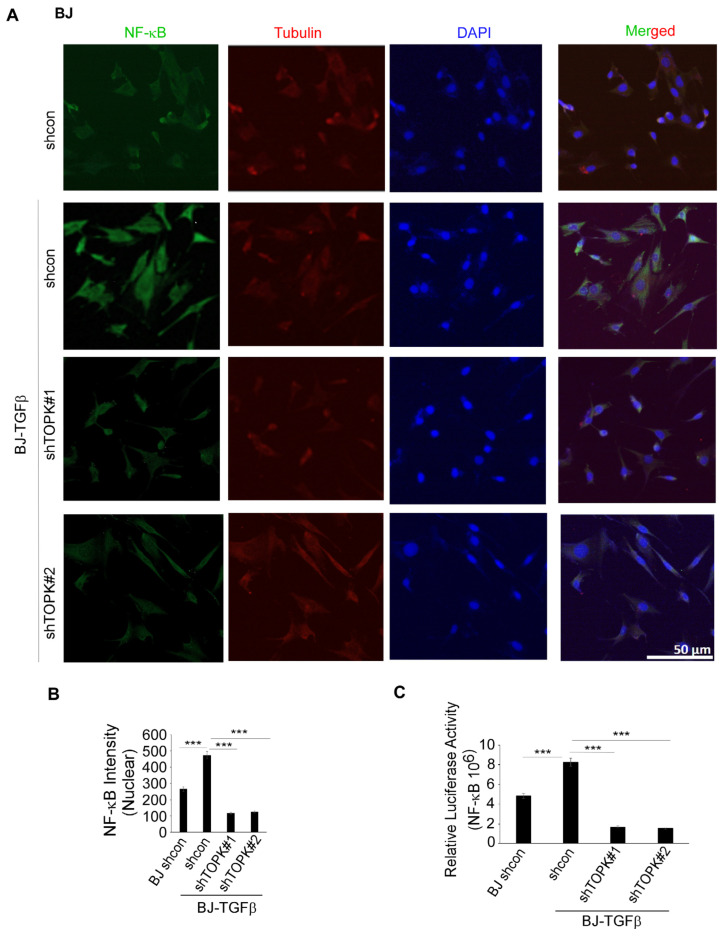
TOPK regulates NF-κB activation and nuclear translocation in BJ-TGFβ cells. (**A**,**B**) TOPK knockdown inhibits NF-κB nuclear translocation in BJ-TGFβ fibroblasts. The nuclear density of NF-κB was quantified for each sample. (**C**) TOPK knockdown significantly reduced NF-κB activation in BJ-TGFβ fibroblasts, as assessed by a luciferase reporter assay. Asterisks indicate significant differences compared to the control group (***, *p* < 0.001; one-way ANOVA followed by Dunnett’s post hoc test).

NFκB is a key transcription factor that regulates IL19 expression, particularly in response to inflammatory signals. Studies have shown that NFκB activation can enhance IL19 expression [40,41]. Inhibition of the NFκB pathway can reduce the release of IL19 [42]. In this study, we conducted immunofluorescence to detect the levels of NFκB and tubulin, the latter of which was used to visualize the cytoskeletal structure and confirm cell morphology. DAPI was used as a nuclear counterstain. Our results demonstrate that TOPK knockdown significantly suppressed SSL-induced NFκB activation and nuclear translocation in HaCaT keratinocytes (Figure 4). Similarly, TOPK knockdown markedly reduced NFκB activation and nuclear translocation in cSCC cells, both with and without TPA treatment for 24 h (Figure 5). Additionally, TOPK knockdown suppressed NFκB activation and nuclear translocation in both normal fibroblasts and activated BJ-TGFβ or NHDF-TGFβ fibroblasts (Figure 6; Supplementary Figure S4). These results demonstrate that TOPK mediates IL19 expression by the NFκB signaling pathway.

### 3.4. Knockdown of TOPK in Fibroblasts Significantly Suppresses cSCC Cell Growth and Migration in 3D Spheroids and Transwell Co-Culture Models

**Figure 7:**
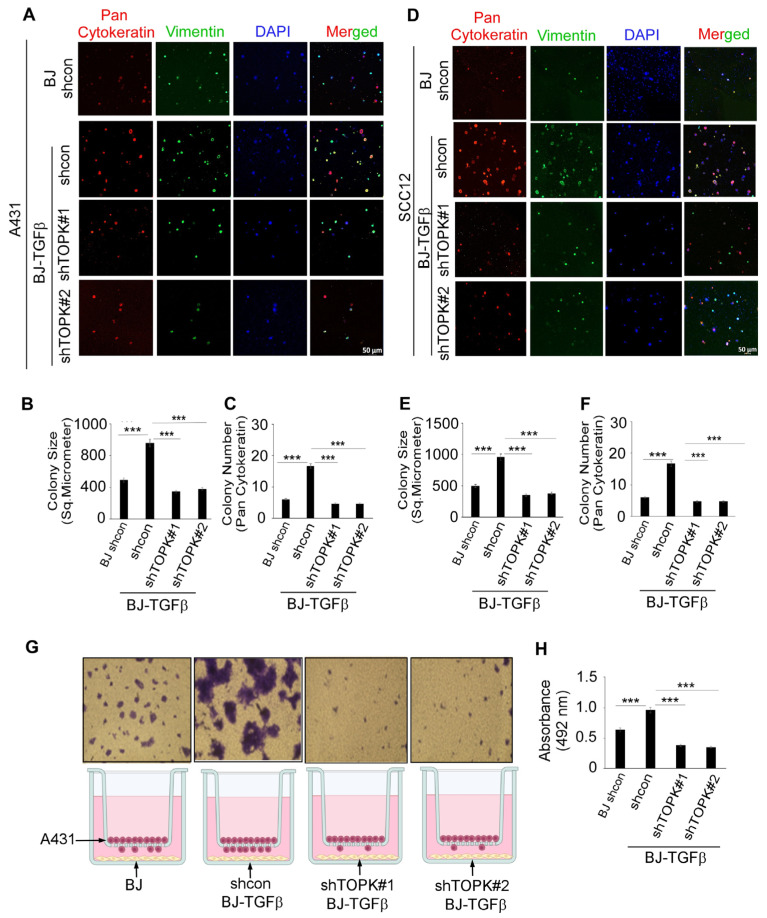
Knockdown of TOPK in fibroblasts suppresses cSCC cell growth and migration in 3D spheroid and Transwell co-culture models. (**A**–**C**) Co-culture of cSCC A431 cells with activated BJ-TGFβ fibroblasts enhanced cSCC growth compared to normal BJ fibroblasts, while TOPK knockdown significantly reduced colony formation size and number. (**D**–**F**) In cSCC SCC12 cells, co-culture with BJ-TGFβ fibroblasts promoted growth, whereas TOPK knockdown suppressed colony formation. (**G**,**H**) In a Transwell co-culture model, activated BJ-TGFβ fibroblasts significantly enhanced cSCC cell migration, while TOPK knockdown effectively reduced migration. Asterisks indicate significant differences compared to the control group (***, *p* < 0.001; one-way ANOVA).

Knockdown of TOPK in BJ-TGFβ cells was assessed in co-culture with cSCC A431 (Figure 7A–C; Supplementary Figure S5) and SCC12 (Figure 7D–F; Supplementary Figure S6). Pan-cytokeratin was used as a marker to identify cSCC cells, while vimentin was selected to label fibroblasts and assess stromal cell phenotype and spatial distribution within the 3D spheroid co-culture system. DAPI was used as a nuclear counterstain. The results demonstrate that activated BJ-TGFβ co-culture enhanced cSCC cell growth compared to normal BJ fibroblasts. TOPK knockdown significantly reduced colony formation size and number in the 3D spheroid co-culture model. Additionally, CM from TOPK-knockdown BJ-TGFβ cells decreased cSCC cell growth compared to the control (Supplementary Figure S7). Furthermore, activated BJ-TGFβ in a Transwell co-culture model significantly promoted cSCC cell migration compared to normal BJ fibroblasts, while TOPK knockdown effectively suppressed cSCC cell migration in this model (Figure 7G,H).

## 4. Discussion

Our study provides novel insights into the role of TOPK in UV-induced skin damage and carcinogenesis, underscoring its potential as a therapeutic target and biomarker for NMSC. Through comprehensive RNA-seq analysis, we identified significant gene expression changes associated with TOPK deletion in WT and knockout mice following solar UV exposure.

Natural daily exposure to solar UV radiation, particularly UVA and UVB, has a cumulative effect on the skin. Even at sub-erythemal levels, chronic low-dose UV exposure can lead to DNA damage, oxidative stress, and immunosuppression, contributing to premature skin aging and increasing the risk of skin carcinogenesis [43,44,45]. Normal skin possesses protective mechanisms such as DNA repair pathways and melanin production; however, repeated daily UV exposure can overwhelm these defenses, resulting in mutagenesis and clonal expansion of damaged keratinocytes [46,47]. This process plays a critical role in the initiation and progression of non-melanoma skin cancers, including cSCC. Therefore, our experimental model using SSL aims to closely mimic the carcinogenic impact of natural sunlight in a controlled and reproducible manner.

Notably, our results demonstrate that TOPK drives IL19 expression, which mediates crosstalk between cSCC cells and fibroblasts. These findings highlight the broad impact of TOPK on multiple cellular pathways and its role in regulating IL19 expression, contributing to the solar UV-induced skin response and carcinogenesis.

TOPK has been previously implicated in several oncogenic processes, including skin cancers [18,19,20]. In this study, we demonstrated that TOPK deletion results in the suppression of key signaling pathways, such as cytokine–cytokine receptor interaction signaling, PI3K/AKT, MAPKs, PKG, cAMP, and calcium signaling (Figure 1 and Figure 2). These pathways are known to be critical for cell proliferation, survival, and inflammatory responses, playing essential roles in tumor initiation and progression. The cytokine–cytokine receptor interaction pathway is particularly significant in regulating immune cell recruitment and tumor-associated inflammation, which are key factors in the tumor microenvironment [48,49]. The PI3K/AKT pathway promotes cell survival, proliferation, and metabolism, with its aberrant activation frequently observed in skin carcinogenesis [50,51,52]. The MAPK signaling cascade, including ERK, JNK, and p38, regulates cellular responses to environmental stressors such as UV radiation, and its dysregulation has been implicated in skin cancer development [53,54]. Additionally, PKG and cAMP signaling contribute to intracellular signaling dynamics that regulate gene transcription, immune modulation, and fibroblast activation [55,56,57]. Calcium signaling is crucial for maintaining cellular homeostasis and intercellular communication, and has been linked to cancer cell progression and the activation of fibroblasts and immune cells in the tumor microenvironment [55,56,57]. The suppression of these pathways in TOPK-deficient cells and tissues suggests that TOPK serves as a central regulator of tumor-promoting signaling cascades in response to UV-induced skin damage. These findings provide strong evidence that targeting TOPK could effectively disrupt multiple oncogenic and inflammatory pathways, reducing the tumorigenic potential of solar UV exposure and highlighting its potential as a therapeutic target in NMSC.

A particularly intriguing finding in our study is the marked suppression of IL19 expression following TOPK deletion. IL19 is a pro-inflammatory cytokine that has been associated with tumor progression and immune modulation in several malignancies. Our results indicate that IL19 expression is significantly reduced in both mouse skin tissues and human cSCC and fibroblast cells upon TOPK knockdown. Functionally, IL19 promotes cSCC cell proliferation and activates major oncogenic pathways [58,59,60], including PI3K/AKT, ERK, and even TOPK itself, suggesting a potential feedforward loop between IL19 and TOPK in NMSC progression. A pivotal revelation of this study is the role of TOPK in mediating bidirectional communication between epidermal keratinocytes, dermal fibroblasts, and cSCC cells, which collectively drive TME remodeling. Solar UV radiation damages both epidermal keratinocytes and dermal fibroblasts, but how these layers cooperatively facilitate carcinogenesis remains poorly understood. Here, we demonstrate that TOPK activation in keratinocytes and fibroblasts generates a cytokine-rich milieu that fuels cSCC progression.

The expression of αSMA and FAPα serves as a hallmark of fibroblast activation into myofibroblasts and CAFs, key drivers of TME remodeling [61]. In the epidermal compartment, UV-induced TOPK activation triggers IL19 secretion from keratinocytes and cSCC cells (Figure 3B,C). IL19 acts as a paracrine signal to dermal fibroblasts, inducing their activation into myofibroblasts and marked by αSMA and FAPα overexpression (Figure 3L–N). Activated myofibroblasts or CAFs, in turn, secrete growth factors and matrix-remodeling enzymes that promote tumor cell survival, proliferation, and migration (Figure 7). This epidermal-to-dermal signaling axis is reinforced by our finding that chronic TGFβ treatment, a hallmark of advanced tumor microenvironments, upregulates IL19 in fibroblasts (Figure 3G), suggesting a feedforward loop where stromal IL19 further amplifies TOPK activity in adjacent tumor cells (Figure 3F). Such reciprocity between TOPK and IL19 creates a self-sustaining circuit that escalates malignancy.

Conversely, dermal fibroblasts expressing TOPK enhance cSCC growth and migration through physical and secretory interactions. In 3D spheroid co-cultures, TOPK knockdown in fibroblasts reduce cSCC colony size and number (Figure 7A–F), likely by attenuating the production of pro-tumorigenic factors (e.g., IL19, matrix metalloproteinases) that normally facilitate tumor cell expansion and ECM remodeling. Additionally, TOPK knockdown in fibroblasts effectively suppresses cSCC cell migration (Figure 7G,H). Previous studies have demonstrated that IL19 plays a critical role in cancer metastasis [62,63]. These results suggest that TOPK may promote metastasis by regulating IL19 expression.” These findings align with clinical observations that CAF density correlates with cSCC aggressiveness, but our work identifies TOPK as the molecular linchpin enabling this collaboration. These findings suggest that IL19 not only promotes cancer cell proliferation but also remodels the tumor microenvironment to facilitate disease progression.

**Figure 8:**
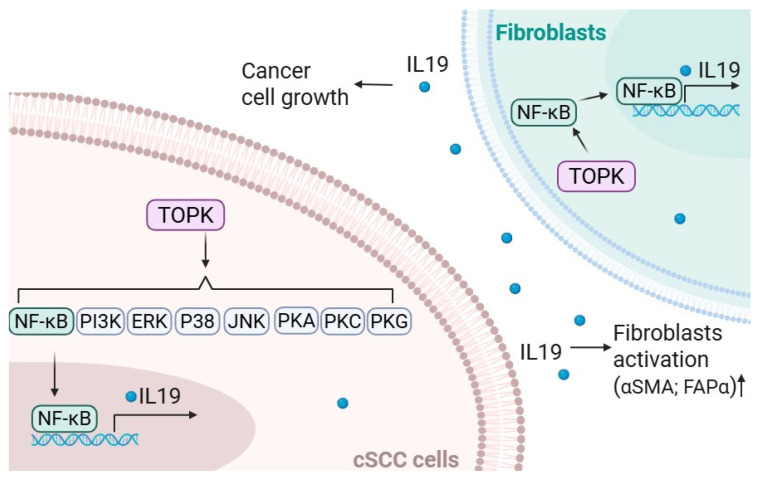
TOPK/IL19 signaling crosstalk between cSCC cells and fibroblasts. In cSCC cells, TOPK activates multiple downstream pathways, including NF-κB, PI3K, ERK, p38, JNK, PKA, PKC, and PKG, which converge to promote the transcription and secretion of IL19. TOPK also enhances the expression of NFκB and IL19 in fibroblasts. Secreted IL19 promotes cSCC cell growth and acts in a paracrine manner on nearby fibroblasts, leading to their activation, as indicated by the increased expression of αSMA and FAPα. These activated fibroblasts further support cancer cell growth, suggesting a feedforward loop that drives tumor progression through IL19-mediated communication between tumor cells and the surrounding stroma.

Overall, our findings establish a mechanistic link between TOPK, IL19, and fibroblast activation in NMSC. The schematic model illustrates how, in cSCC cells, TOPK activates multiple signaling pathways to induce IL19 expression and secretion. Secreted IL19, in turn, promotes cancer cell growth and acts in a paracrine manner on adjacent fibroblasts, enhancing their activation, as evidenced by the increased αSMA and FAPα expression. Activated fibroblasts then contribute further to tumor progression, establishing a feedforward loop of tumor–stroma communication mediated by the TOPK–IL19 axis (Figure 8). The interplay between these factors highlights a potential therapeutic axis that could be exploited for skin cancer prevention and treatment. Targeting TOPK and IL19 simultaneously may provide a more effective strategy to inhibit both tumor growth and the supportive tumor microenvironment. Future studies should focus on developing specific inhibitors of TOPK and evaluating their efficacy in preclinical models of skin cancer.

## 5. Conclusions

In summary, our study elucidates the pivotal role of TOPK in solar UV-induced skin damage and NMSC progression. By modulating key oncogenic pathways and promoting IL19 expression, TOPK contributes to a pro-tumorigenic microenvironment that supports both cancer cell proliferation and fibroblast activation. These findings underscore the therapeutic potential of targeting TOPK and IL19 in the treatment and prevention of NMSC.

## Data Availability

The datasets used and/or analyzed during the current study are available from the corresponding author upon reasonable request.

## Supplementary Materials

The following supporting information can be downloaded at: https://www.mdpi.com/article/10.3390/cancers17132067/s1. Supplementary data for Figure 1; Supplementary data for Figure 2; Supplementary Figures (Supplementary Figure S1–S7); Supplementary Figure Legends; Supplementary Methods.

## Abbreviations

The following abbreviations are used in this manuscript:

NMSC	Non-melanoma skin cancers
BCC	Basal cell carcinoma
cSCC	Cutaneous squamous cell carcinoma
UV	Solar ultraviolet
AK	Actinic keratosis
TOPK	T-LAK cell-originated protein kinase
TME	Tumor microenvironment
CAF	Cancer-associated fibroblast
IL19	Interleukin-19
NHDF	Normal human dermal fibroblast
SSL	Solar-simulated light
TGFβ	Transforming Growth Factor-β
αSMA	α-smooth muscle actin
FAPα	Fibroblast activation protein α
CM	Conditioned media
