# Supplementary material for: TOPK Drives IL19-Mediated Crosstalk Between Cancer Cells and Fibroblasts to Promote Solar UV-Induced Skin Damage and Carcinogenesis
Source: Cancers (Basel). 2025 Jun 20;17(13):2067. doi: 10.3390/cancers17132067 (PMC12248498; doi:10.3390/cancers17132067)
Supplement: Supplementary file 1 [file cancers-17-02067-s001.zip › Supplementary Figure Legends.pdf]

Supplementary Figure Legends

Figure S1. Knockdown of TOPK suppresses kinase phosphorylation in SCC12 cells shTOPK and shCon SCC12 cell lines were generated, and the phosphorylation levels and total expression of kinases were analyzed by Western blotting.

Figure S2. TOPK knockdown significantly suppresses IL19 expression and secretion in both normal NHDF fibroblasts and activated NHDF-TGFβ fibroblasts. (A) IL19 and αSMA expression were elevated in chronic TGFβ treated BJ-TGFβ fibroblasts compared with normal BJ fibroblasts. **(B, C)** shTOPK and shCon NHDF or NHDF-TGFβ cell lines were generated. The expression level of TOPK was evaluated by Western blotting. **(D, E)** TOPK knockdown significantly suppressed IL19 expression and secretion in both normal NHDF fibroblasts and activated NHDF-TGFβ fibroblasts.

Figure S3. A larger version of the immunotolerance model from Figure 3M for better visualization.

Figure S4. Knockdown of TOPK suppresses NF-κB nuclear translocation. TOPK knockdown inhibits NF-κB nuclear translocation in NHDF-TGFβ fibroblasts. The nuclear density of NF-κB was quantified for each sample. Asterisks indicate significant differences compared to the control group (***, $p < 0.001$; Dunnett's test, one-way ANOVA).

Figure S5. A larger version of the immunotolerance model from Figure 5A for better visualization.

Figure S6. A larger version of the immunotolerance model from Figure 5D for better visualization.

Figure S7. TOPK mediates fibroblast secretions that modulate cSCC cell growth. (A, B) Conditioned medium (CM) from shTOPK BJ-TGFβ fibroblasts suppressed A431 and SCC12 cell growth, as assessed by MTS and crystal violet staining assays. **(C, D)** Similarly, CM from shTOPK NHDF-TGFβ fibroblasts inhibited A431 and SCC12 cell growth, as determined by MTS and crystal violet staining assays. Asterisks indicate significant differences compared to the control group (**, $p < 0.01$; ***, $p < 0.001$; Dunnett's test, one-way ANOVA).
