# Supplementary material for: TOPK Drives IL19-Mediated Crosstalk Between Cancer Cells and Fibroblasts to Promote Solar UV-Induced Skin Damage and Carcinogenesis
Source: Cancers (Basel). 2025 Jun 20;17(13):2067. doi: 10.3390/cancers17132067 (PMC12248498; doi:10.3390/cancers17132067)
Supplement: Supplementary file 1 [file cancers-17-02067-s001.zip › Supplementary Figures.pdf]

Figure S1

SCC12

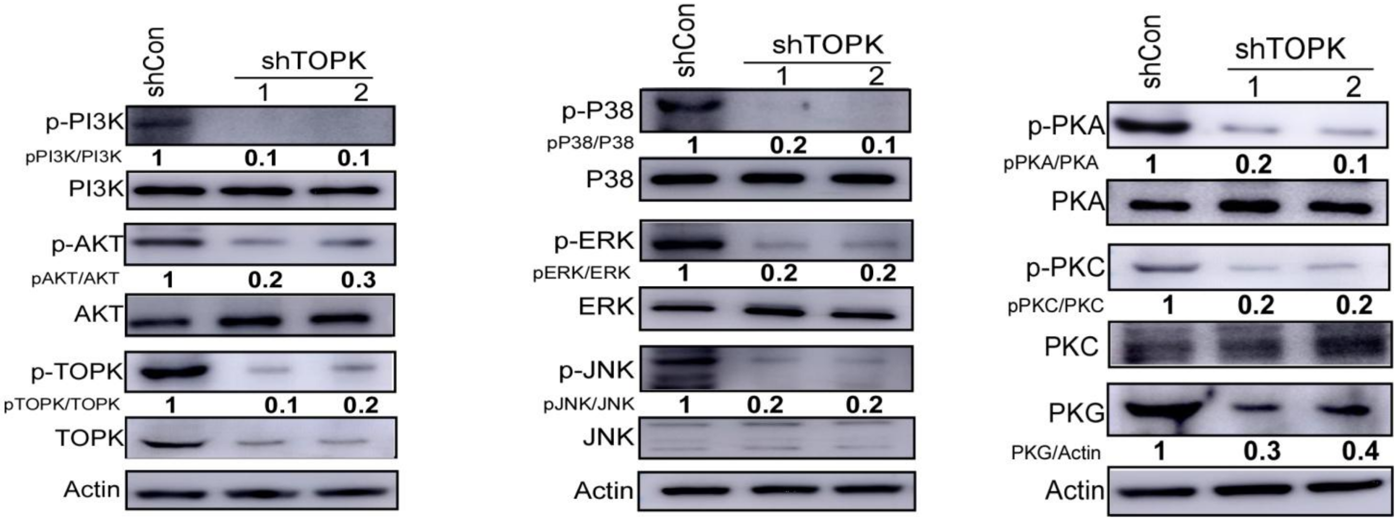

Figure S2

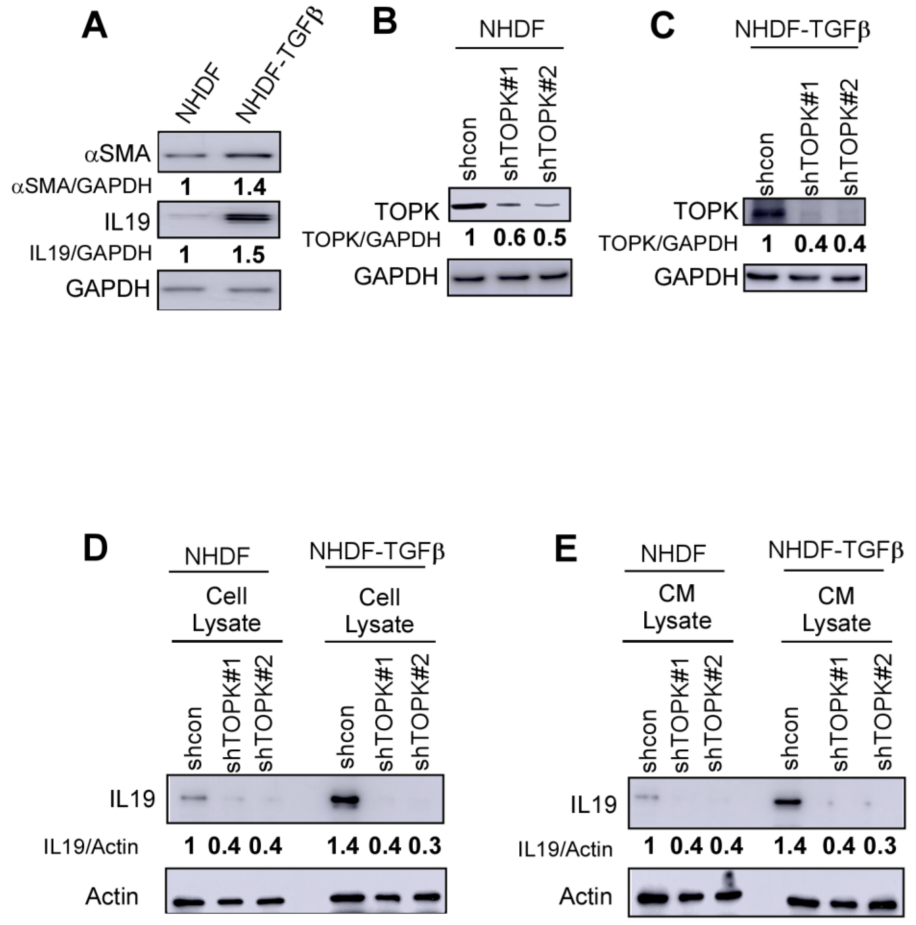
A
NHDF
NHDF-TGFβ
αSMA
αSMA/GAPDH
1
1.4
IL19
IL19/GAPDH
1
1.5
GAPDH
B
NHDF
shcon
shTOPK#1
shTOPK#2
TOPK
TOPK/GAPDH
1
0.6
0.5
GAPDH
C
NHDF-TGFβ
shcon
shTOPK#1
shTOPK#2
TOPK
TOPK/GAPDH
1
0.4
0.4
GAPDH
D
NHDF
NHDF-TGFβ
Cell Lysate
Cell Lysate
shcon
shTOPK#1
shTOPK#2
shcon
shTOPK#1
shTOPK#2
IL19
IL19/Actin
1
0.4
0.4
1.4
0.4
0.3
Actin
E
NHDF
NHDF-TGFβ
CM Lysate
CM Lysate
shcon
shTOPK#1
shTOPK#2
shcon
shTOPK#1
shTOPK#2
IL19
IL19/Actin
1
0.4
0.4
1.4
0.4
0.3
Actin

Figure S3

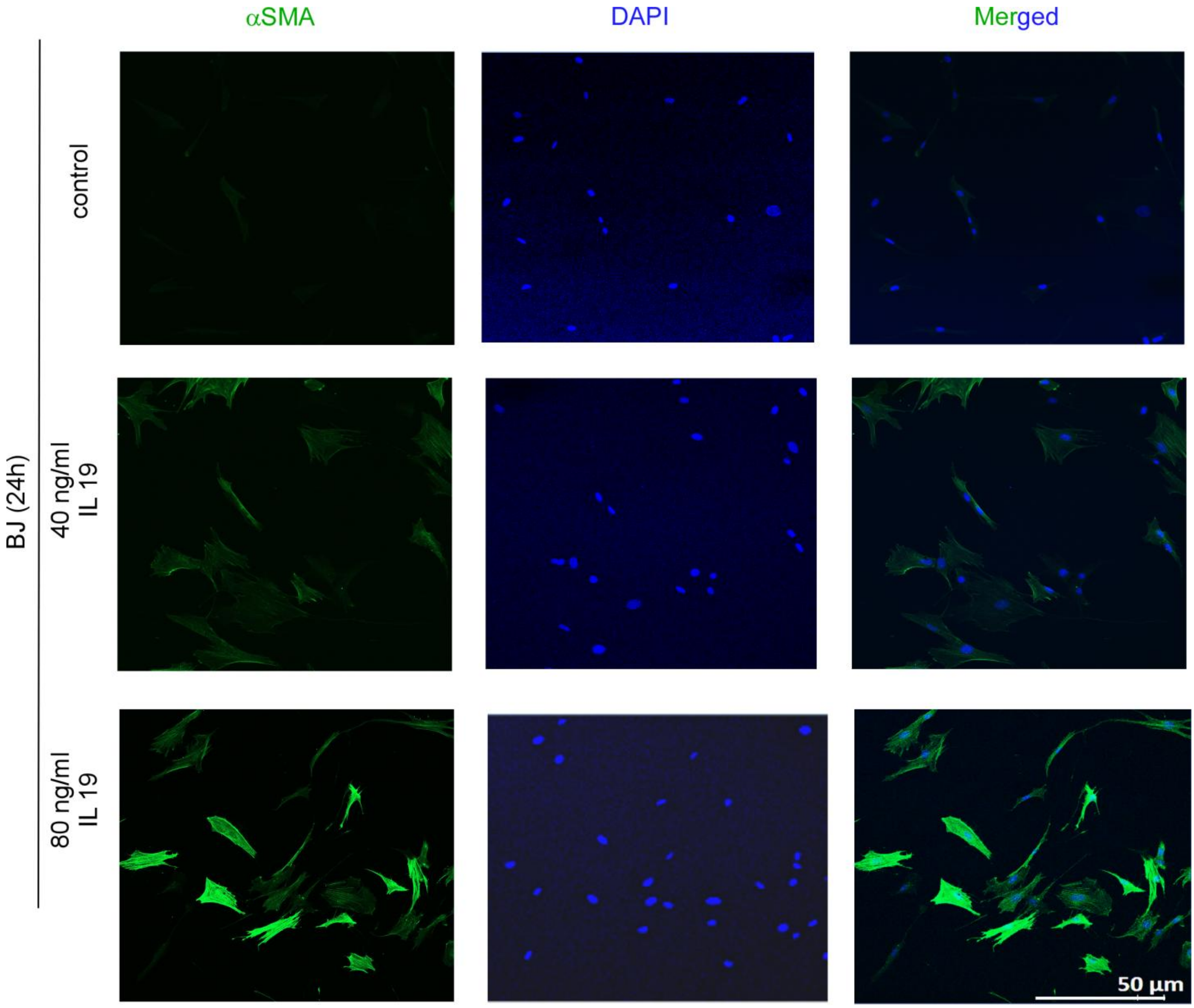

αSMA
DAPI
Merged
BJ (24h)
control
40 ng/ml
IL 19
80 ng/ml
IL 19
50 µm

Figure S4

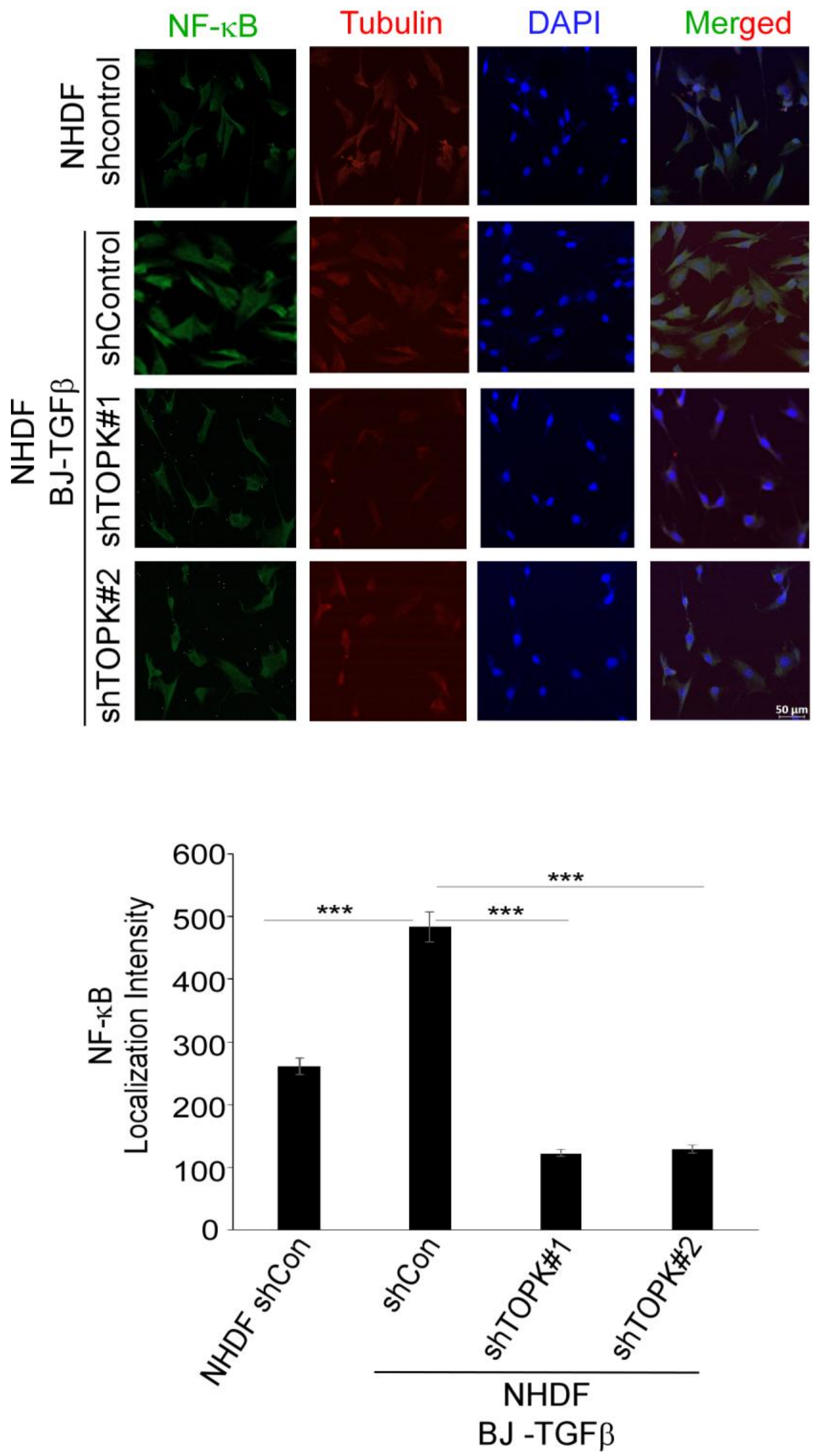
NF-κB
Tubulin
DAPI
Merged
NHDF shcontrol
NHDF BJ-TGFβ
shControl
shTOPK#1
shTOPK#2
50 µm
600
500
400
300
200
100
0
NF-κB Localization Intensity
***
***
***
NHDF shCon
shCon
shTOPK#1
shTOPK#2
NHDF
BJ -TGFβ

Figure S5

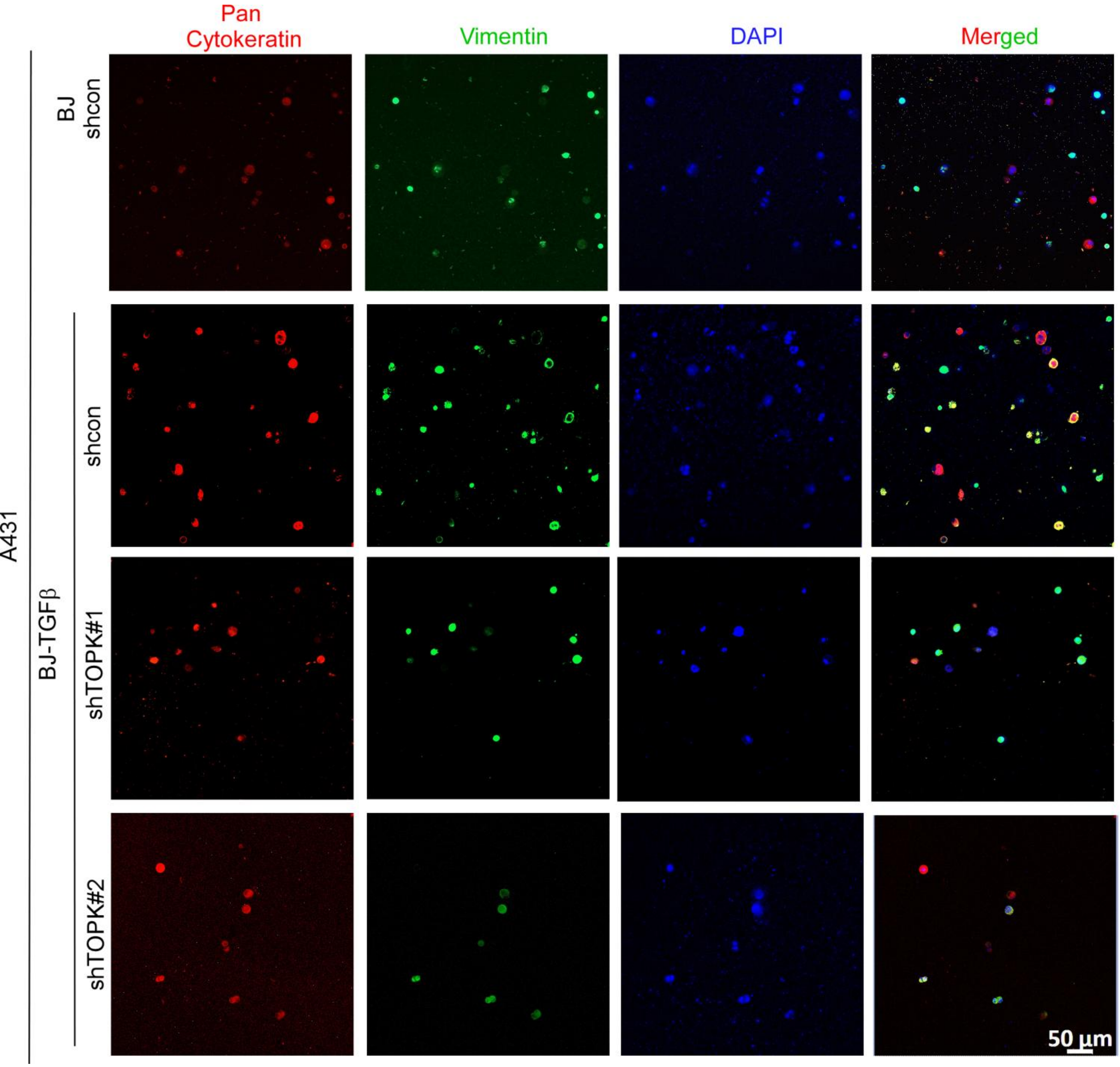

Pan Cytokeratin
Vimentin
DAPI
Merged
A431
BJ
shcon
BJ-TGFβ
shcon
shTOPK#1
shTOPK#2
50 μm

Figure S6

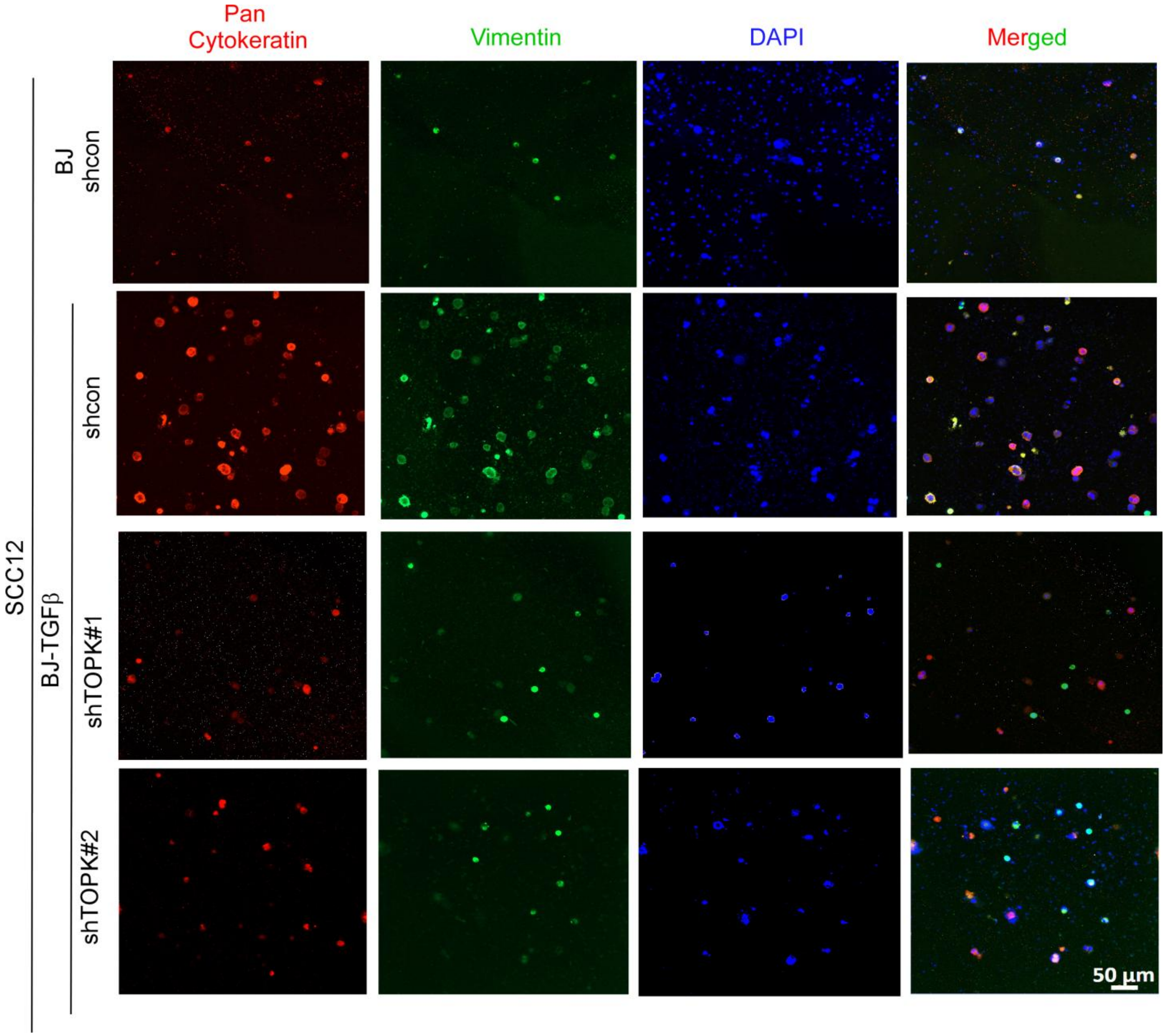
Pan Cytokeratin
Vimentin
DAPI
Merged
BJ shcon
shcon
shTOPK#1
shTOPK#2
BJ-TGFβ
SCC12
50 μm

Figure S7

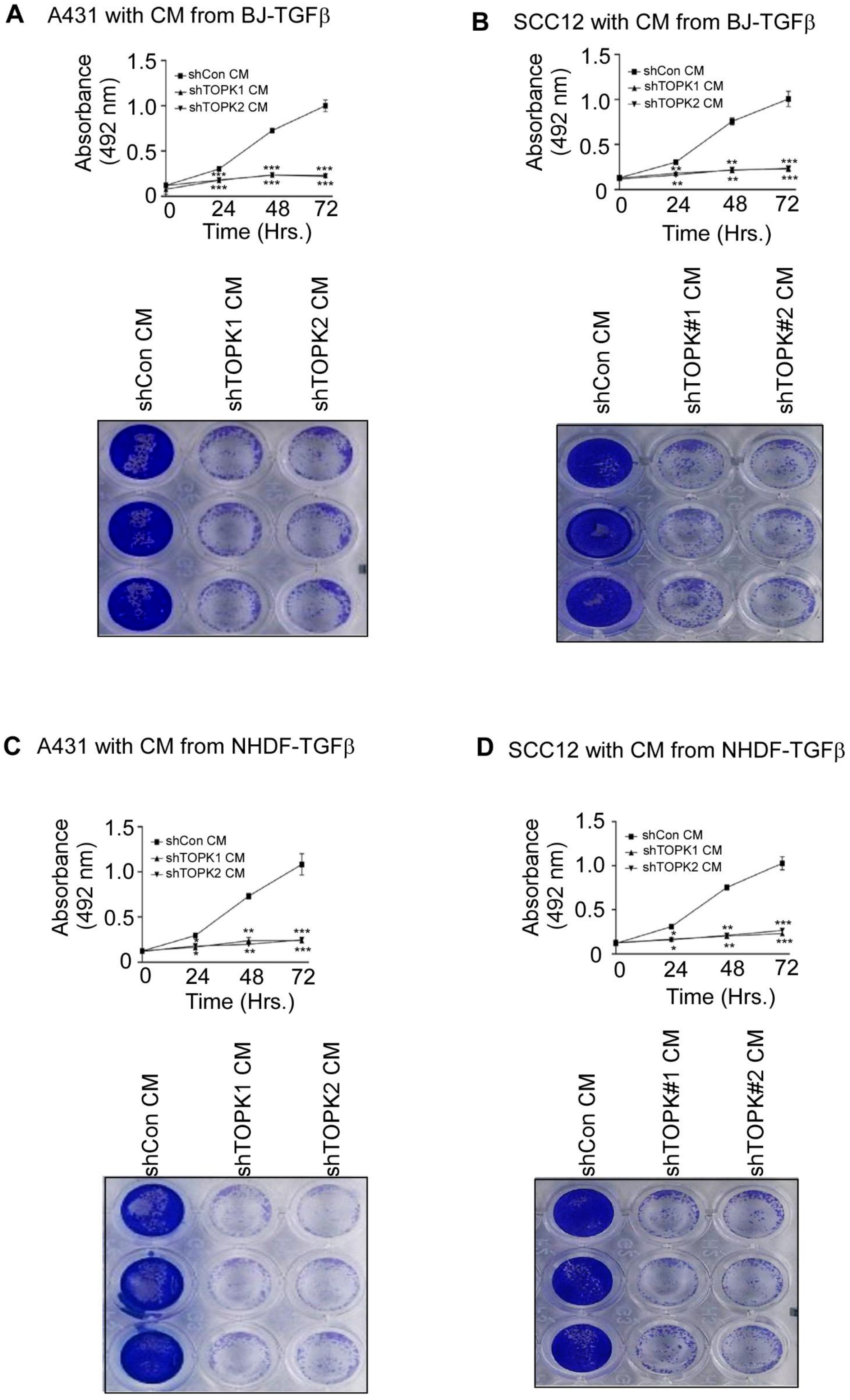
A A431 with CM from BJ-TGFβ
Absorbance (492 nm)
Time (Hrs.)
shCon CM
shTOPK1 CM
shTOPK2 CM
B SCC12 with CM from BJ-TGFβ
Absorbance (492 nm)
Time (Hrs.)
shCon CM
shTOPK1 CM
shTOPK2 CM
shCon CM
shTOPK#1 CM
shTOPK#2 CM
C A431 with CM from NHDF-TGFβ
Absorbance (492 nm)
Time (Hrs.)
shCon CM
shTOPK1 CM
shTOPK2 CM
D SCC12 with CM from NHDF-TGFβ
Absorbance (492 nm)
Time (Hrs.)
shCon CM
shTOPK1 CM
shTOPK2 CM
shCon CM
shTOPK#1 CM
shTOPK#2 CM
