# Supplementary material for: TOPK Drives IL19-Mediated Crosstalk Between Cancer Cells and Fibroblasts to Promote Solar UV-Induced Skin Damage and Carcinogenesis
Source: Cancers (Basel). 2025 Jun 20;17(13):2067. doi: 10.3390/cancers17132067 (PMC12248498; doi:10.3390/cancers17132067)
Supplement: Supplementary file 1 [file cancers-17-02067-s001.zip › SupplemetaryMethods.pdf]

RNA extraction library construction and sequencing

Total RNA was extracted using the RNeasy Mini QIAcube Kit (QIAGEN, 19300 Germantown, MD) following the manufacturer's instructions and purity was checked by using the Bioanalyzer 2100 and RNA 6000 Nano LabChip Kit (Agilent, Santa Clara, CA). After total RNA extraction and purification, mRNA was extracted using Dynabeads Oligo (dT) (Thermo Fisher, Waltham, CA). Following purification, the mRNA was fragmented using the Magnesium RNA Fragmentation Module (Cat. e6150, NEB, Ipswich, MA). From the RNA fragments, cDNA by SuperScript™ II Reverse Transcriptase (Cat. 1896649, Invitrogen, Carlsbad, CA) was synthesized. Dual-index adapters were ligated to the fragments, and size selection was performed with Amperex beads. After the heat-labile UDG enzyme (Cat.m0280, NEB) treatment of the U-labeled second-stranded DNAs, the ligated products were amplified with PCR. We then performed the 2×150 bp paired-end sequencing (PE150) on an Illumina Novaseq™ 6000 following the vendor's recommended protocol. The RNA sequencing was performed by LC Science (Houston, TX).

Bioinformatics analysis

Sequence and filtering of clean reads

A cDNA library was constructed using pooled RNA from mouse skin samples and sequencing was performed using the Illumina NovaseqTM 6000 sequence platform. Using the Illumina paired-end RNA-seq approach, we sequenced the transcriptome, generating a total of 2 million x 150 bp paired-end reads. Reads obtained from the sequencing analysis included adapters or low-quality bases that may have the potential to affect the assembly and analysis. Thus, to obtain high quality clean reads, reads were further filtered by Cutadapt

(https://cutadapt.readthedocs.io/en/stable/,version:cutadapt-1.9 [1] The parameters were as follows: 1) removing reads containing adapters; 2) removing reads containing polyA and polyG; 3) removing reads containing more than 5% of unknown nucleotides (N); 4) removing low quality reads containing more than 20% of low quality (Q-value ≤20) bases (http://www.bioinformatics.babraham.ac.uk/projects/fastqc/,0.11.9) , including the Q20, Q30 and GC-content of the clean data. Following that procedure, a total of G bp of cleaned, paired-end reads were produced.

Alignment with reference genome

We aligned reads of all samples to the < research species > reference genome by using the HISAT2 (https://daehwankimlab.github.io/hisat2/,version:hisat2-2.0.4) package, which initially removes a portion of the reads based on quality information accompanying each read and then maps the reads to the reference genome. HISAT2 allows multiple alignments per read (up to 20 by default) and a maximum of two mismatches when mapping the reads to the reference. HISAT2 builds a database of potential splice junctions and confirms these by comparing the previously unmapped reads against the database of putative junctions [2-4].

Quantification of gene abundance

The mapped reads of each sample were assembled using StringTie (http://ccb.jhu.edu/software/stringtie/,version:stringtie-1.3.4d) with default parameters. Then, all transcriptomes from all samples were merged to reconstruct a comprehensive transcriptome by using gffcompare software (http://ccb.jhu.edu/software/stringtie/gffcompare.shtml,version:gffcompare-0.9.8). After the final

transcriptome was generated, StringTie and ballgown (http://www.bioconductor.org/packages/release/bioc/html/ballgown.html) were used to estimate the expression levels of all transcripts and perform expression abundance for mRNAs by calculating FPKM (fragment per kilobase of transcript per million mapped reads) value [4-6].

Differentially expressed genes analysis

We utilized the R package Limma (version 3.40.6) for differential analysis, aiming to identify differences in gene expression between the treatment and control groups. The genes with a parameter of p value below 0.05 and absolute fold change ≥ 2 were considered differentially expressed genes. Then we performed the enrichment analysis (KEGG) by using the National Institutes of Health DAVID Bioinformatics software (https://david.ncifcrf.gov/tools.jsp).

Gene expression omnibus (GEO) data analysis

We obtained four datasets (GSE2503, GSE45164, GSE42677, and GSE45216) from the Gene Expression Omnibus (GEO), which we merged using the R software package inSilicoMerging. We used the Empirical Bayes method to adjust for batch effects in the microarray expression data. Next, we used the R software package Limma (version 3.40.6) to identify differentially expressed genes between actinic keratosis (AK) or squamous cell carcinoma (SCC) and normal skin ($p < 0.05$, and absolute fold change ≥ 2).

Western blot analysis

HaCaT cells (1×10^6) were seeded in 10-cm dishes. Once the cells reached 60–70% confluence, they were briefly rinsed with 1× PBS. Fresh DMEM containing 10% FBS and a 1% antibiotic

solution were then added 5 hours before exposure to solar-simulated light (SSL; 60 kJ/m^2 UVA and 2.9 kJ/m^2 UVB). Following SSL exposure, the cells were incubated at 37°C in a humidified 5% CO_2 incubator for designated time points.

SCC-12 and A431 cells (1×10^6) were similarly cultured in 10-cm dishes, with the medium changed after 48 hours. BJ and Normal Human Dermal Fibroblasts (NHDF) fibroblasts were seeded at 60–70% confluence in 60-mm round tissue culture dishes with serum-containing medium. The following day, fibroblasts were incubated in serum-starved medium containing IL-19 at 40 ng/mL or 80 ng/mL for 24 hours at 37°C.

For protein extraction from conditioned medium, 50 g of trichloroacetic acid (TCA) were dissolved in 100 mL of distilled water. A 5-mL sample of conditioned medium was collected, mixed with an equal volume of TCA, vortexed to resuspend, and incubated on ice for 30 minutes. The mixture was then centrifuged at 13,000 rpm for 30 minutes at 4°C. The supernatant fraction was discarded, and the pellet was washed twice with 1 mL of ice-cold acetone. The samples were centrifuged at 13,000 rpm for 10 minutes at 4°C, the supernatant fraction was removed, and the pellet was air-dried. After drying, the pellet was resuspended in 250 μL of RIPA lysis buffer, followed by sonication.

Protein lysates from cells and conditioned medium were prepared using 1× RIPA cell lysis buffer supplemented with a protease and phosphatase inhibitor cocktail (R4100-010, Gene Depot, Katy, Texas). Protein quantification was performed using a standard curve method, and absorbance was measured with the Multiscan MCC Plate Reader 355 Microplate Reader (Thermo Fisher Scientific, Alexandria, VA).

Equal amounts of protein samples were resolved by SDS-PAGE and transferred onto polyvinylidene difluoride (PVDF) membranes (EMD Millipore, Billerica, MA). Membranes

were blocked with 5% nonfat milk for 1 hour at room temperature and then incubated overnight at 4°C with primary antibodies (1:1000), following the manufacturer's recommended dilutions. A horseradish peroxidase (HRP)-conjugated secondary antibody (1:10,000) was used for detection. Protein lysates from cells and conditioned medium were run on the same gel. GAPDH was used as a loading control. Protein bands were visualized using chemiluminescent reagents and detected with the Amersham Imager 600 RGB (GE Healthcare Biosciences, Marlborough, MA).

References

1. Martin, M., Cutadapt removes adapter sequences from high-throughput sequencing reads. *EMBnet. journal* **2011,** 17, (1), 10-12.
2. Kim, D.; Paggi, J. M.; Park, C.; Bennett, C.; Salzberg, S. L., Graph-based genome alignment and genotyping with HISAT2 and HISAT-genotype. *Nature biotechnology* **2019,** 37, (8), 907-915.
3. Kim, D.; Langmead, B.; Salzberg, S. L., HISAT: a fast spliced aligner with low memory requirements. *Nature methods* **2015,** 12, (4), 357-360.
4. Pertea, M.; Kim, D.; Pertea, G. M.; Leek, J. T.; Salzberg, S. L., Transcript-level expression analysis of RNA-seq experiments with HISAT, StringTie and Ballgown. *Nature protocols* **2016,** 11, (9), 1650-1667.
5. Kovaka, S.; Zimin, A. V.; Pertea, G. M.; Razaghi, R.; Salzberg, S. L.; Pertea, M., Transcriptome assembly from long-read RNA-seq alignments with StringTie2. *Genome biology* **2019,** 20, (1), 1-13.

6. Pertea, M.; Pertea, G. M.; Antonescu, C. M.; Chang, T.-C.; Mendell, J. T.; Salzberg, S. L., StringTie enables improved reconstruction of a transcriptome from RNA-seq reads. *Nature biotechnology* **2015,** 33, (3), 290-295.
